# Characteristics of New Onset Herpes Simplex Keratitis after Keratoplasty

**DOI:** 10.1155/2018/4351460

**Published:** 2018-10-22

**Authors:** Xiaolin Qi, Miaolin Wang, Xiaofeng Li, Yanni Jia, Suxia Li, Weiyun Shi, Hua Gao

**Affiliations:** Shandong Eye Hospital, Shandong Eye Institute, Shandong Academy of Medical Sciences, Jinan, China

## Abstract

**Purpose:**

To observe clinical characteristics and treatment outcomes of new onset herpes simplex keratitis (HSK) after keratoplasty.

**Methods:**

Among 1,443 patients (1,443 eyes) who underwent keratoplasty (excluding cases of primary HSK) in Shandong Eye Hospital, 17 patients suffered postoperative HSK. The clinical manifestations, treatment regimens, and prognoses of the patients were evaluated.

**Results:**

The incidence of new onset HSK after keratoplasty was 1.18%. Epithelial HSK occurred in 10 eyes, with dendritic epithelial infiltration in 6 eyes and map-like epithelial defects in 4 eyes. Nine eyes had lesions at the junction of the graft and recipient. Stromal necrotic and endothelial HSK occurred in 7 eyes, presenting map-shaped ulcers in the entire corneal graft and recipient (two eyes) or at the graft-recipient junction (five eyes). Confocal microscopy revealed infiltration of a large number of dendritic cells at the junction of the lesion and transparent cornea. All 10 eyes with epithelial lesions and two eyes suffering stromal lesions of ≤1/3 corneal thickness healed after systematic and local antiviral treatment. Best-corrected visual acuity and corneal graft transparency were restored. For stromal HSK with an ulcer of >1/3 corneal thickness, amniotic membrane transplantation was performed, and visual acuity and graft transparency decreased significantly.

**Conclusion:**

New onset HSK after keratoplasty primarily resulted in epithelial and stromal lesion, involving both the graft and recipient. Effective treatments included antiviral medications and amniotic membrane transplantation. Delayed treatment may lead to aggravated graft opacification.

## 1. Introduction

It is possible that keratoplasty induces herpes simplex keratitis (HSK), resulting in epithelial defects or ulcers in the grafts, even if the patient has no history of viral keratitis [1–4]. The majority of related studies have just been case reports with no systematic reports of clinical characteristics, typing, and diagnosis or treatment. The diagnosis of HSK is usually difficult, and this viral infection is often confused with infectious ulcers or immunological rejection of the corneal graft, leading to inappropriate use of antiinfective drugs or glucocorticoids, thereby adversely affecting or even exacerbating the disease. To provide more information about the clinical diagnosis and management, we retrospectively analyzed clinical data from patients with new onset HSK after keratoplasty and evaluated the clinical characteristics, diagnoses, and treatment methods.

## 2. Materials and Methods

### 2.1. Patients

This study was approved by the Institutional Review Board of Shandong Eye Hospital and adhered to the tenets of the Declaration of Helsinki. Clinical data of 1,443 patients who underwent keratoplasty (excluding cases of primary HSK) from January 2013 to January 2017 in our hospital with a regular follow-up of more than 1 year were retrospectively analyzed. HSK was identified in 17 patients (17 eyes) during the postoperative follow-up. The diagnostic criteria of HSK included (1) subjective symptoms of the affected eye, including redness and swelling, paresthesia, photophobia, tearing, and decreased visual acuity; (2) typical eye signs like dendritic or map-like infiltration or ulcers, terminal expansion, positive fluorescein sodium staining, discoid edema of the stroma, and substantial keratic precipitates (KPs); (3) negative results of corneal scraping smear examination for bacteria and fungi and laser confocal microscopy for fungal hyphae and amoeba cysts; and (4) gradual improvement of the patient after systemic and local antiviral medications [5–7].

### 2.2. Outcome Measures

The medical history included symptoms associated with the patient's complaint, the time of HSK onset, medication, changes in disease condition, and recurrence. Best-corrected visual acuity (BCVA), intraocular pressure (IOP), typical eye signs, corneal graft transparency, and epithelial integrity were recorded. In vivo confocal microscopy (HRT3; Heidelberg Engineering, Dossenheim, Germany) was used to observe epithelial and stromal inflammatory changes of the corneal graft and the distribution of dendritic cells. RTVue optical coherence tomography (OCT; Optovue, Fremont, California, USA) was used to clarify the depth of corneal stromal lesions.

## 3. Results

The incidence of new onset HSK after keratoplasty was 1.18% during the follow-up period of 38 ± 14.7 months (range, 10–55 months). The patients were 10 males and 7 females, aged 4–67 years (mean, 51 ± 15.1 years). The primary lesions included bacterial corneal ulcers in 8 eyes, immune-related corneal ulcers in 4 eyes, corneal leucoma in 2 eyes, fungal corneal ulcers in 2 eyes, and keratoconus in 1 eye. The surgical treatment was penetrating keratoplasty for 10 eyes and anterior lamellar keratoplasty for 7 eyes. All donor corneal tissues were provided by Shandong Red Cross Eye Bank.

All 17 patients denied repeated symptoms of redness, photophobia, tearing, decreased vision, and other symptoms of HSK. The time interval between keratoplasty and HSK onset was 11.7 ± 9 months, with the earliest case occurring at 1 month postoperatively. The onset time was postoperative 1–3 months for 2 eyes, 3–6 months for 3 eyes, 6–12 months for 6 eyes, 12–18 months for 4 eyes, and 18–24 months for 2 eyes.

### 3.1. Clinical Manifestations

Sudden eye swelling, pain with rubbing, photophobia, tearing, decreased visual acuity, and other discomforts in the affected eye were noted by 15 patients. The average duration from the onset to seeking treatment was 5.4 ± 1.6 days (3.9 ± 1.3 days for epithelial lesions and 7.9 ± 2.6 days for stromal necrotic lesions).

The BCVA of the 17 eyes decreased after the onset of HSK by 2 lines in eight eyes, 3–4 lines in four eyes, and 5–6 lines in five eyes. The IOP was within the normal range in all eyes.

**Figure 1:**
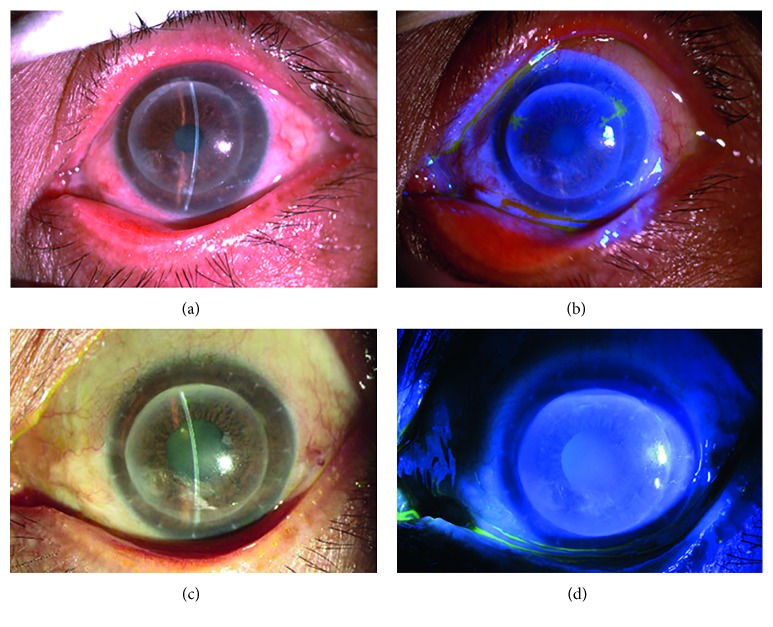
Slit lamp examination of patient 1 with epithelial HSK. (a) Two years after deep anterior lamellar keratoplasty for corneal leucoma. (b) Dendritic epithelial infiltration, terminal expansion, and positive fluorescein sodium staining at 2 and 10o'clock and the junction of the graft and recipient. (c) Epithelial infiltration disappearance after systemic and local antiviral treatment. (d) Negative fluorescein sodium staining.

**Figure 2:**
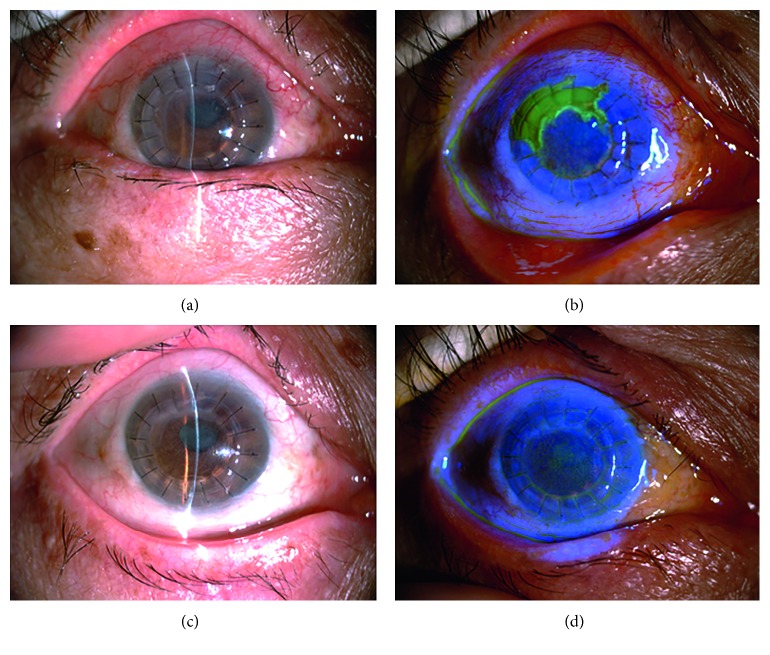
Slit lamp examination of patient 2 with epithelial HSK. (a) Two months after penetrating keratoplasty for bacterial corneal ulcers. (b) Map-shaped fluorescein sodium staining at the junction of the graft and recipient between 9 and 1o'clock. (c) The healed epithelium and clear cornea after systemic and local antiviral treatment. (d) Negative fluorescein sodium staining.

HSK in 10 eyes was epithelial type. Six eyes had dendritic epithelial infiltration, terminal expansion, and positive fluorescein sodium staining with no obvious edema of the graft (Figure 1). Among them, the lesion was located in the center of the graft in one eye and at the junction of the graft and recipient in five eyes. The other four eyes had mild edema in the corneal graft with map-like epithelial defects, terminal expansion, and positive fluorescein sodium staining (Figure 2). Defective areas were located at the junction of the graft and recipient between 4 and 6o'clock, with an area of approximately 4 × 6 mm^2^. The endothelial cell layer had a relatively large amount of finely pigmented KPs.

**Figure 3:**
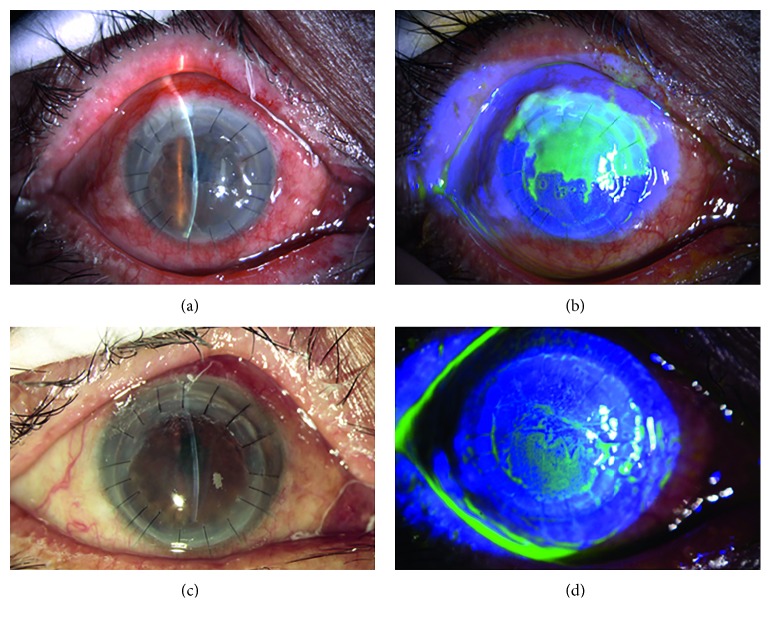
Slit lamp examination of patient 3 with stromal necrotic and endothelial HSK. (a) The edematous corneal graft at 6 months after penetrating keratoplasty for fungal corneal ulceration. (b) Map-shaped ulceration at the junction of the graft and recipient between 10 and 3o'clock. (c) The healed ulcer and clear cornea after systemic and local antiviral treatment. (d) Negative fluorescein sodium staining.

**Figure 4:**
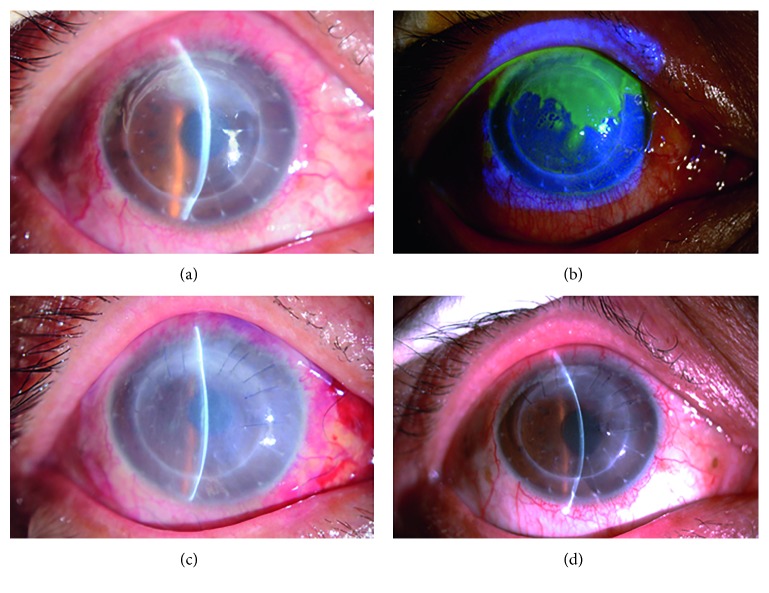
Slit lamp examination of patient 4 with stromal necrotic and endothelial HSK. (a) The edematous corneal graft at one and a half years after penetrating keratoplasty for bacterial corneal ulceration. (b) Map-shaped ulceration at the junction of the graft and recipient between 10 and 2o'clock. (c) The ulcer healing after amniotic membrane transplantation. (d) The corneal clarity was decreased.

**Figure 5:**
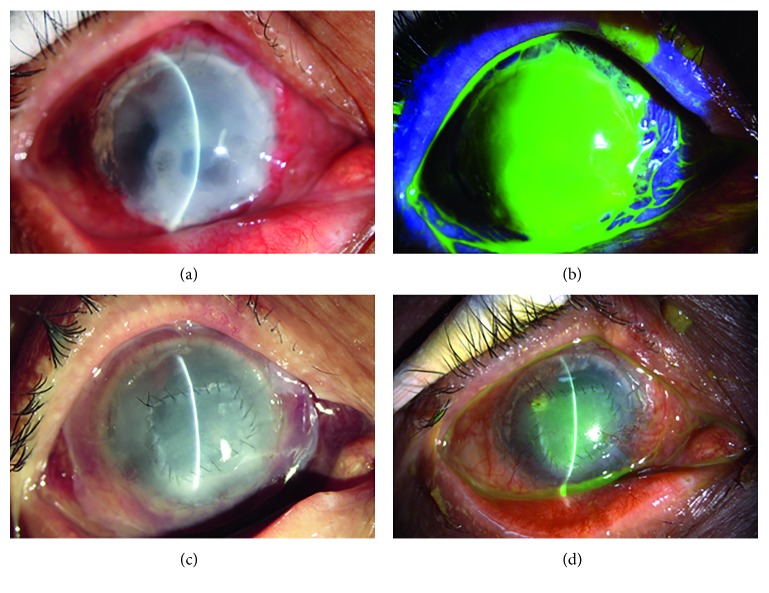
Slit lamp examination of patient 4 with stromal necrotic and endothelial HSK. (a) Corneal graft edema and opacity at one and a half years after penetrating keratoplasty for bacterial corneal ulceration. (b) Fluorescein sodium staining displaying ulceration of the entire corneal graft involving the whole transplant bed. (c) The ulcer healing after double amniotic membrane transplantation. (d) The residual amniotic membrane at the final follow-up. The corneal clarity was decreased significantly.

Stromal necrotic and endothelial HSK was found in seven eyes, presenting with edema in the corneal graft and map-shaped ulcers. Five eyes had lesions located at the junction of the graft and recipient between 4 and 6o'clock, with an area of approximately 4 × 8 mm^2^; the endothelial cell layer had a relatively large amount of finely pigmented KPs (Figures 3 and 4). Two of them displayed ulcers covering the entire corneal graft and involving the whole transplant bed (Figure 5).

Moreover, anterior chamber reaction was graded as +1 flare and +1 cell. No HSK recurred after medical or surgical management.

### 3.2. In Vivo Confocal Microscopy

**Figure 6:**
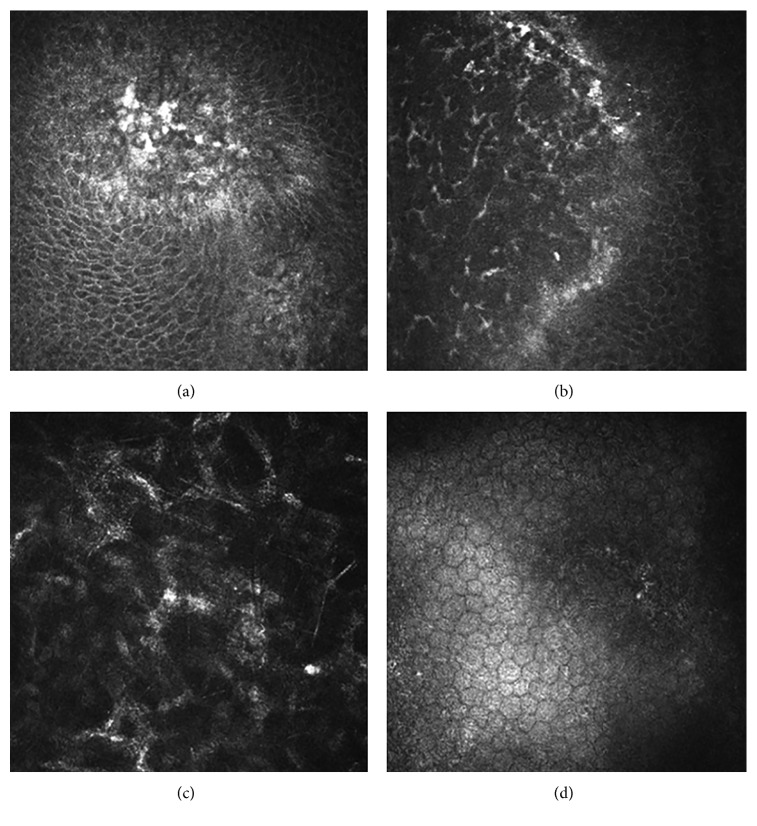
In vivo confocal microscopy examination of epithelial HSK. (a) The epithelial cells were swollen and necrotic. (b) A large number of dendritic cells were observed at the junction of the lesion and transparent cornea. (c) The polygonal cells of the stromal cells in the superficial stromal layer showed enhanced reflectivity and were arranged in a cross-hatched pattern. (d) Little KPs were observed in the endothelial cell layer.

In the eyes with epithelial HSK, the epithelial cells in the lesions were swollen and necrotic. Few or no subbasal nerve plexuses were observed. A large number of dendritic cells and basal epithelial cells formed netting via long interdigitating dendrites. The anterior elastic layer was damaged. The polygonal cells of the stromal cells in the superficial stromal layer showed enhanced reflectivity and were arranged in a cross-hatched pattern, but most nuclei were not visible. Little KPs were observed in the endothelial cell layer (Figure 6).

**Figure 7:**
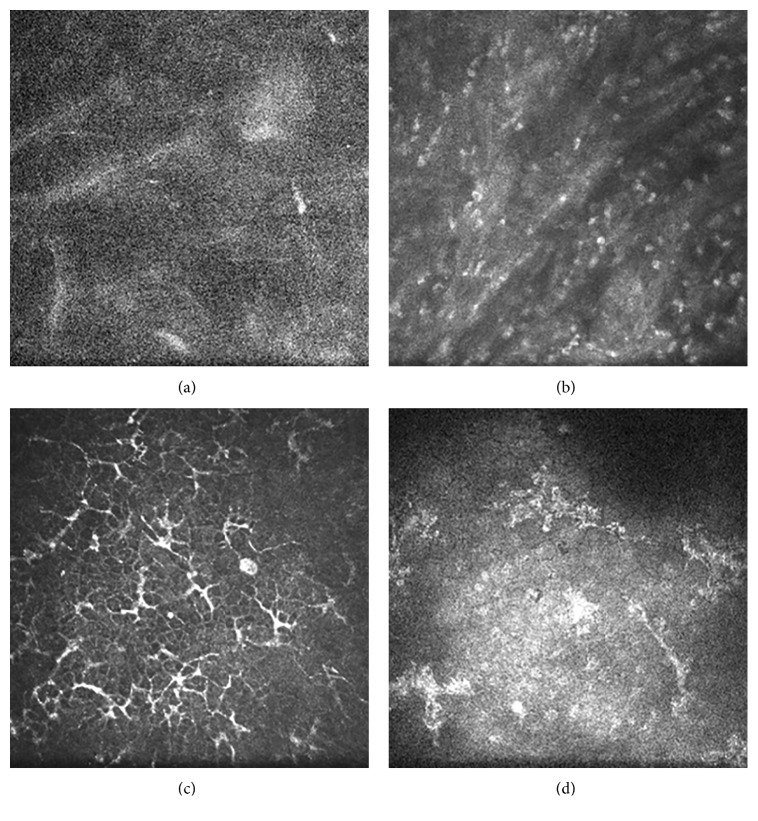
In vivo confocal microscopy examination of stromal necrotic and endothelial HSK. (a) Scanning images of the stromal cells were unclear. (b) A large amount of inflammatory cells were observed. (c) A large number of dendritic cells were observed, and wire netting was formed via long interdigitating dendrites at the junction of the lesion and transparent cornea. (d) Lots of KPs were observed in the endothelial cell layer.

For the stromal necrotic and endothelial type, scanning images of the stromal cells in the lesions were unclear, showing a large amount of inflammatory cell infiltration. At the junction of the lesion and transparent cornea, however, a large number of dendritic cells, basal epithelial cells, and subbasal nerve plexuses were observed to form netting via long interdigitating dendrites. Lots of KPs were present in the endothelial cell layer (Figure 7).

### 3.3. Rtvue OCT

Corneal OCT was mainly used to identify the depth of the stromal lesions. Among the 7 eyes with stromal necrosis, 2 eyes had an ulcer ≤1/3 corneal thickness and 5 eyes had an ulcer >1/3 corneal thickness. In the two eyes with the entire corneal graft affected, the ulcer depth was up to 1/2 corneal thickness.

### 3.4. Treatment and Outcomes

Systemic and local antiviral therapy was administered in all 17 patients. Acyclovir was given intravenously (5 mg/kg) every 8 hours for 7 days, and then Aciclovir Tablets (3 × 400 mg) were given orally for 3 months. Moreover, 0.1% acyclovir eye drops (Wuhan Wujing Pharmaceutical Co., Wuhan, China) were used every 2 hours. For cases of epithelial HSK, the use of glucocorticoids was prohibited. For cases of stromal and endothelial HSK, 0.1% fluorometholone eye drops (Santen, Osaka, Japan), tobramycin dexamethasone eye drops (Alcon, Puurs, Belgium), and tobramycin dexamethasone eye ointment (Alcon, Puurs, Belgium) were employed based on disease conditions.

In all eyes with epithelial type lesions, epithelial infiltration disappeared after systemic and local antiviral treatment and was resolved within an average of 3.2 ± 1.9 days (Figures 1 and 2). The BCVA was restored to the level before disease onset with a transparent corneal graft.

For patients with stromal necrotic lesions, the treatment options and the prognoses were closely related to the depth of the ulcers. The ulcers in two eyes with an ulcer ≤1/3 corneal thickness healed after systemic and local antiviral treatment, with an average healing time of 7.5 days, and the BCVA was restored to the level before HSK onset (Figure 3). In the five patients with an ulcer >1/3 corneal thickness, the ulcers did not heal after systemic and local antiviral treatment. Two patients with the entire corneal graft involved refused to undergo a second keratoplasty; therefore, double amniotic membrane transplantation was performed, and these ulcers healed after 18.5 days (Figure 5). The patients exhibited a BCVA of 3–4 lines less than that before disease onset, decreased transparency of the corneal grafts, and scars at the ulcer healing sites. The remaining 3 patients underwent amniotic membrane transplantation with an average healing time of 12.3 days (Figure 4). They exhibited a BCVA of 1–2 lines less than that before disease onset, decreased transparency of the corneal grafts, and scars at the ulcer healing sites.

## 4. Discussion

Recurrent herpetic keratitis is a leading cause of infectious blindness in the world [8, 9]. Ophthalmic surgery, especially keratoplasty, can induce the onset of HSK, even if the patient has never had symptoms of herpes simplex virus infection prior to surgery [10–12]. Due to its low incidence, many physicians lack adequate understanding of the clinical manifestations of HSK. Misdiagnosis and inappropriate medical therapy may lead to treatment errors or delays so that the patient's vision and corneal graft transparency are affected. In this study, we evaluated the clinical characteristics and treatment outcomes of new onset HSK after keratoplasty in patients who had no prior HSK.

The lesion location of new onset HSK after keratoplasty was found to be mostly at the graft-recipient junction, and even at the entire corneal graft and surrounding transplant bed. This can be used to distinguish HSK from other corneal infections, because the other infectious graft ulcers, except for peripheral ulcers caused by loose sutures, are often located in the center of the graft and rarely involve the adjacent transplant bed. In the current study, most patients complained of decreased visual acuity within a short time, accompanied by conjunctival hyperemia and corneal edema, which made the physicians first suspect corneal graft rejection. The following 2 points may help to distinguish HSK from corneal graft rejection. First, in new onset HSK after keratoplasty, we observed typical dendritic or map-like epithelial infiltration, which may lead to stromal ulcers in severe cases and involve the adjacent transplant bed. Conversely, the immunological rejection of a corneal graft primarily manifests as edema of the corneal graft, an endothelial rejection line, neovascularization filling, and engorgement. Moreover, graft rejection is rarely associated with epithelial damage [13–15]. Second, in vivo confocal microscopy is a powerful tool that can assist in making a diagnosis [16–18]. The dendritic cells did not aggregate at the center of the corneal lesion but at the junction of the lesion area and transparent cornea in the new onset HSK cases [19]. In cases of corneal graft rejection, large numbers of dendritic cells and infiltration are only available in the area covering the epithelial or endothelial rejection line [20–22].

Fifteen of 17 patients complained of sudden discomfort with redness and decreased vision, but the degree of severity was related to whether they received treatment in time. The patients with timely treatment experienced mild, primarily epithelial type corneal lesions, with an average time of treatment of 3.9 ± 1.3 days; otherwise, the disease condition gradually worsened, leading to the formation of a map-like corneal ulcer after 7.9 ± 2.6 days, which can spread to the entire cornea. In all cases of epithelial lesions, epithelial infiltration disappeared quickly after antiviral treatment, with an average cure time of 3.2 ± 1.9 days, and corneal graft transparency was restored. The treatment option and prognoses of patients with stromal lesions were closely related to the depth of the ulcer. When the ulcer depth was ≤1/3 corneal thickness, the average healing time was 7.5 days after antiviral treatment, but corneal graft transparency and BCVA were not affected. Once the depth was >1/3 corneal thickness, the ulcers were not liable to heal after medication, and amniotic membrane transplantation or double amniotic membrane transplantation was required, with an average healing time extended to 18.5 days. After the surgical intervention, the patients exhibited scarring at the site of the healing ulcer, significantly decreasing corneal graft transparency and BCVA. Early diagnosis and standardized treatment may provide the greatest possible resolution of pathological changes; a lack of knowledge of the disease or delayed treatment can cause irreversible damage to vision and corneal graft transparency.

Herpes simplex virus-1 (HSV-1) can establish latent infections in the trigeminal ganglia [23]. HSV-1 DNA is also detected in the corneas of humans and animals with quiescent HSK, suggesting that the cornea might be another latency site of HSV-1 [24]. In patients without a clinical history of HSK, the emergence of HSV-1 DNA in corneas with primary graft failure suggested donor-recipient transmission through keratoplasty [25–27]. In our study, keratoplasty can induce the onset of HSK. The HSV infection may arise from any of three main mechanisms: intracorneal multiplication of virus after reactivation in sensitive ganglia, reactivation of latent-state virus in the residual cornea of the recipient, or through reactivation of a donor-recipient transmission virus [28]. But it is difficult to confirm the source of virus because donor corneas are not routinely screened for HSV-1 in our eye bank, which is the main limitation of this retrospective study. In addition, all 17 patients were considered as no episodes of HSK previously by means of limited medical records and their denial of repeated symptoms, with a lack of immunohistochemistry or polymerase chain reaction (PCR) assay, which is another limitation of this retrospective study. Improved detection methods and additional screening of donor corneas for HSV-1 may provide an improved understanding of corneal latency and its role in primary graft failure [29].

To conclude, the results of this study showed that HSK occurred in 1.18% of patients after keratoplasty. The diagnosis of HSK was mainly based on typical eye signs, such as dendritic epithelial infiltration and map-like epithelial lesions or ulcers, involving both the graft and adjacent recipient. Early stage lesions were primarily epithelial type, which could be cured quickly with antiviral treatment without affecting vision or corneal graft transparency. Delayed treatment may aggravate disease conditions and require amniotic membrane transplantation and even corneal transplantation, causing irreversible damage to vision and corneal graft transparency.

## 5. Conclusions

Keratoplasty can induce the onset of herpes simplex keratitis, resulting in epithelial defects or ulcers both in the graft and recipient. Effective treatments included antiviral medications and amniotic membrane transplantation. Delayed treatment causes irreversible damage to vision and corneal graft transparency.

## Data Availability

The data used to support the findings of this study are included within the article.
